# Supplementary material for: Content-rich biological network constructed by mining PubMed abstracts
Source: BMC Bioinformatics. 2004 Oct 8;5:147. doi: 10.1186/1471-2105-5-147 (PMC528731; doi:10.1186/1471-2105-5-147)
Supplement: Additional File 2 — The original results of the above study (non-essential files are deleted to keep the file size under the limit set by BMC bioinformatics). [file 1471-2105-5-147-S2.bz2 › chilibotAdditionalFile2/dip30/301ID7486683E1292/html/PLCG1_PDGFRB.html]

 


 **PLCG1** and **PDGFRB** 
  
Found 24 abstracts in PubMed, retrieved 24.  
 

 What does Google say? 
 PDF only 
| .edu only 

---

**Interactive relationship** (e.g. stimulation, inhibition, etc)

**Neutral relationship**- The phosphorylated C terminal tail of the PDGFR  [ **PDGFRB** ]  bound PLC gamma 1  [ **PLCG1** ] , but did not associate with phosphatidylinositol PI 3 kinase or GTPase activating protein GAP.  Ref: 7689724 Oncogene, 1993
- Here we show that functional phospholipase C gamma1  [ **PLCG1** ]  PLC gamma1  [ **PLCG1** ]  is required for c Abl activation by PDGFR  [ **PDGFRB** ] .  Ref: 12652307 Nat Cell Biol, 2003
- These results indicate that phosphorylation at Tyr 1021 in the tail of the PDGFR  [ **PDGFRB** ]  creates a specific binding site for PLC gamma 1  [ **PLCG1** ] .  Ref: 7689724 Oncogene, 1993
- Identification of residues in the beta platelet derived growth factor receptor  [ **PDGFRB** ]  that confer specificity for binding to phospholipase C gamma 1  [ **PLCG1** ] .  Ref: 7689724 Oncogene, 1993
- Upon ligand induced tyrosine phosphorylation, the platelet derived growth factor PDGF receptor PDGFR  [ **PDGFRB** ]  beta subunit associates with PLC gamma 1  [ **PLCG1** ] , RasGAP, P13K, and a 64 kd protein.  Ref: 7682895 Cell, 1993
- On ligand induced tyrosine phosphorylation, PDGFR  [ **PDGFRB** ]  associated with phospholipase C PLC gamma 1  [ **PLCG1** ] , Ras GTPase activating protein RasGAP, and phosphatidylinositol 3 kinase PI3K but not with the Syp growth factor receptor bound protein 2 Son of Sevenless complex.  Ref: 9612285 Am J Physiol, 1998
- Our previous studies indicated that PI3K and PLC  [ **PLCG1** ]  gamma were required for relay of the mitogenic signal of beta PDGFR  [ **PDGFRB** ] .  Ref: 7760802 Mol Cell Biol, 1995
- Thus, it appears that phosphorylation of the PDGFR  [ **PDGFRB** ]  at Y 1021 is required for the stable association of PLC  [ **PLCG1** ]  gamma to the receptor s carboxy terminus, the production of inositol phosphates, and initiation of the maximal mitogenic response.  Ref: 7678051 Mol Cell Biol, 1993
- The activated beta PDGFR  [ **PDGFRB** ]  physically associated and phosphorylated signaling molecules such as ras GTPase activating protein GAP and phospholipase C gamma PLC  [ **PLCG1** ]  gamma.  Ref: 7489253 Arterioscler Thromb Vasc Biol, 1995
- Exposure of immunoblots containing bacterially expressed GAP or PLC  [ **PLCG1** ]  gamma to activated alpha or beta PDGF receptors led to a comparable high affinity binding of each receptor to PLC  [ **PLCG1** ]  gamma, while the beta PDGFR  [ **PDGFRB** ]  showed a 5 fold higher binding affinity for GAP.  Ref: 8387491 J Biol Chem, 1993
- We constructed and characterized a series of PDGFR  [ **PDGFRB** ]  mutants which contain binding sites for PI3K as well as one additional protein, either RasGAP, SHP 2, or PLC  [ **PLCG1** ]  gamma.  Ref: 8816504 Mol Cell Biol, 1996
- Binding of platelet derived growth factor PDGF to the PDGF receptor PDGFR  [ **PDGFRB** ]  beta subunit triggers receptor tyrosine phosphorylation and the stable association of a number of signal transduction molecules, including phospholipase C gamma PLC  [ **PLCG1** ]  gamma, the GTPase activating protein of ras GAP, and phosphatidylinositol 3 kinase PI3K.  Ref: 7678051 Mol Cell Biol, 1993
- Upon binding of platelet derived growth factor PDGF, the PDGF beta receptor PDGFR  [ **PDGFRB** ]  undergoes autophosphorylation on distinct tyrosine residues and binds several SH2 domain containing signal relay enzymes, including phosphatidylinositol 3 kinase PI3K, phospholipase C gamma PLC  [ **PLCG1** ]  gamma, the GTPase activating protein of Ras RasGAP, and the tyrosine phosphatase SHP 2.  Ref: 8816504 Mol Cell Biol, 1996
- The PDGF stimulated release of G6PD was partially restored in PDGFR  [ **PDGFRB** ]  mutants in which either phosphatidylinositol 3 kinase or phospholipase C gamma 1  [ **PLCG1** ]  could associate with the PDGFR  [ **PDGFRB** ] .  Ref: 8182086 J Biol Chem, 1994
- Autophosphorylation of beta PDGFR  [ **PDGFRB** ]  and tyrosine phosphorylation of PLC  [ **PLCG1** ]  gamma, PI3Kp85 and Shc were detected only in PDGF BB stimulated cells that express beta PDGFR  [ **PDGFRB** ] .  Ref: 12700668 Oncogene, 2003

**Non-interactive relationship** (e.g. studied together, co-existance, homology, etc.)

- F5 PDGFR  [ **PDGFRB** ]  failed to activate PLC gamma 1  [ **PLCG1** ] , P13K, or Ras and was unable to trigger DNA synthesis.  Ref: 7682895 Cell, 1993
- P< ; 0.0l, but the expression of PDGFR  [ **PDGFRB** ]  beta and PLC  [ **PLCG1** ]  activities did not show significant changes P>0.05.  Ref: 12589410 Sheng Li Xue Bao, 2001
- Substitution of the proline at the 3 position relative to Tyr 1021 with methionine Y1021IIP >Y1021IIM in the phosphorylated PDGFR  [ **PDGFRB** ]  tail did not alter PLC gamma 1  [ **PLCG1** ]  association, but conferred binding activity towards PI 3 kinase, indicating that this residue is critical in discriminating between PLC gamma 1  [ **PLCG1** ]  and PI 3 kinase.  Ref: 7689724 Oncogene, 1993
- To identify the phosphotyrosine involved in association of phospholipase C PLC gamma 1  [ **PLCG1** ]  with the beta platelet derived growth factor receptor  [ **PDGFRB** ]  PDGFR  [ **PDGFRB** ] , and to investigate which contiguous residues confer specificity for PLC gamma 1  [ **PLCG1** ] , phosphotyrosine containing glutathione S transferase GST fusion proteins possessing different regions of the beta PDGFR  [ **PDGFRB** ]  were incubated with lysates of Rat 2 cells that overexpress PLC gamma 1  [ **PLCG1** ] .  Ref: 7689724 Oncogene, 1993
- Since tyrosine phosphorylation of PLC  [ **PLCG1** ]  gamma enhances its enzymatic activity, we speculated that PDGFR  [ **PDGFRB** ]  mutants that failed to activate PLC  [ **PLCG1** ]  gamma were unable to mediate its tyrosine phosphorylation.  Ref: 7678051 Mol Cell Biol, 1993
- Focusing on the PLC  [ **PLCG1** ]  gamma dependent branch of beta PDGFR  [ **PDGFRB** ]  signaling, we constructed a series of mutant beta PDGFRs that contained the binding sites for pairs of the receptor associated proteins PLC  [ **PLCG1** ]  gamma and PI3K, PLC  [ **PLCG1** ]  gamma and GAP, or PLC  [ **PLCG1** ]  gamma and Syp.  Ref: 7760802 Mol Cell Biol, 1995
- Permitting association of F5 PDGFR  [ **PDGFRB** ]  with either PLC gamma 1  [ **PLCG1** ]  or P13K restored Ras activation and a mitogenic response.  Ref: 7682895 Cell, 1993
- The protein levels of PKC alpha and PLC gamma1  [ **PLCG1** ]  were unchanged, but those of PLD1 and PDGFR  [ **PDGFRB** ]  were reduced with age.  Ref: 12086695 Exp Gerontol, 2002
- A PDGFR  [ **PDGFRB** ]  mutant Y1021 that binds and activates phospholipase C gamma PLC  [ **PLCG1** ]  gamma, but not PI 3 kinase, also caused the PDGF dependent translocation of PKC epsilon.  Ref: 8552594 Proc Natl Acad Sci U S A, 1996
- The PDGFR  [ **PDGFRB** ]  mutants that failed to associate with PLC  [ **PLCG1** ]  gamma were not able to mediate the PDGF dependent production of inositol phosphates.  Ref: 7678051 Mol Cell Biol, 1993
- In comparison with the sham group, ACE activity, PLC  [ **PLCG1** ]  activity and the level of PDGFR  [ **PDGFRB** ]  beta were increased by 420.2 percent.  Ref: 12589410 Sheng Li Xue Bao, 2001
- PDGF stimulated the association of PDGFR  [ **PDGFRB** ]  beta with p85, ras GTPase activating protein and PLC  [ **PLCG1** ]  gamma.  Ref: 10697503 Anticancer Res< MedlineTA>Anticancer Res, 2001
- Likewise, phosphorylation and association of GAP and PLC  [ **PLCG1** ]  gamma with PDGF BB activated beta PDGFR  [ **PDGFRB** ]  were unaffected.  Ref: 7489253 Arterioscler Thromb Vasc Biol, 1995
- Thus, the production of inositol phosphates requires not only PLC  [ **PLCG1** ]  gamma tyrosine phosphorylation but also its association with the PDGFR  [ **PDGFRB** ] .  Ref: 7678051 Mol Cell Biol, 1993
- Activities of angiotensin converting enzyme ACE and aorta phospholipase C PLC  [ **PLCG1** ]  were tested, and levels of platelet derived growth factor receptor beta  [ **PDGFRB** ]  PDGFR  [ **PDGFRB** ]  beta and Galphaq 11 protein were measured by Western blot analysis.  Ref: 12589410 Sheng Li Xue Bao, 2001
- PDGF receptor PDGFR  [ **PDGFRB** ] , protein kinase C alpha PKC alpha, phospholipase C gamma1  [ **PLCG1** ]  PLC gamma1  [ **PLCG1** ] , and PLD1 were examined by Western blot analysis.  Ref: 12086695 Exp Gerontol, 2002
- Association of PDGFR  [ **PDGFRB** ]  with PLC gamma 1  [ **PLCG1** ]  and RasGAP followed by their tyrosine phosphorylation failed to activate PLC gamma 1  [ **PLCG1** ] , protein kinase C PKC, and Ras.  Ref: 9612285 Am J Physiol, 1998
- Progressive conversion of the three residues C terminal to Tyr 1021 to the consensus for PI 3 kinase binding YMDM allowed PI 3 kinase association, but did not block PLC gamma 1  [ **PLCG1** ]  binding, suggesting that additional residues other than the three residues immediately following the phosphotyrosine may contribute to the association of PLC gamma 1  [ **PLCG1** ]  with the PDGFR  [ **PDGFRB** ] .  Ref: 7689724 Oncogene, 1993
- Characterization of PDGFR  [ **PDGFRB** ]  mutants in which these phosphorylation sites are substituted with phenylalanine F indicated that Y 1021 and Y 1009 were required for the stable association of PLC  [ **PLCG1** ]  gamma and a 64 kDa protein, respectively.  Ref: 7678051 Mol Cell Biol, 1993
- SB, in the absence of PDGF BB, caused neither beta PDGFR  [ **PDGFRB** ]  tyrosine phosphorylation nor phosphorylation and association of GAP and PLC  [ **PLCG1** ]  gamma with beta PDGFR  [ **PDGFRB** ] .  Ref: 7489253 Arterioscler Thromb Vasc Biol, 1995
- Thus, GAP functions to suppress signal relay by the beta PDGFR  [ **PDGFRB** ] , and it does so by silencing PLC  [ **PLCG1** ]  gamma.  Ref: 7760802 Mol Cell Biol, 1995
